# Acceleration of knee magnetic resonance imaging using a combination of compressed sensing and commercially available deep learning reconstruction: a preliminary study

**DOI:** 10.1186/s12880-023-00962-2

**Published:** 2023-01-09

**Authors:** Hiroyuki Akai, Koichiro Yasaka, Haruto Sugawara, Taku Tajima, Masaru Kamitani, Toshihiro Furuta, Masaaki Akahane, Naoki Yoshioka, Kuni Ohtomo, Osamu Abe, Shigeru Kiryu

**Affiliations:** 1 Department of Radiology, Institute of Medical Science, University of Tokyo, 4-6-1 Shirokanedai, Minato-ku, Tokyo, 108-8639 Japan; 2 Present Address: Department of Radiology, International University of Health and Welfare Narita Hospital, 852 Hatakeda, Narita, Chiba 286-0124 Japan; 3 Department of Radiology, Graduate School of Medicine, University of Tokyo, 7-3-1 Hongo, Bunkyo-ku, Tokyo, 113-8655 Japan; 4 Department of Radiology, International University of Health and Welfare Mita Hospital, 1-4-3 Mita, Minato-ku, Tokyo, 108-8329 Japan; 5 International University of Health and Welfare, 2600-1 Kiakanemaru, Ohtawara, Tochigi 324-8501 Japan

**Keywords:** Artificial intelligence, Deep learning, Magnetic resonance imaging, Knee

## Abstract

**Purpose:**

To evaluate whether deep learning reconstruction (DLR) accelerates the acquisition of 1.5-T magnetic resonance imaging (MRI) knee data without image deterioration.

**Materials and methods:**

Twenty-one healthy volunteers underwent MRI of the right knee on a 1.5-T MRI scanner. Proton-density-weighted images with one or four numbers of signal averages (NSAs) were obtained via compressed sensing, and DLR was applied to the images with 1 NSA to obtain 1NSA-DLR images. The 1NSA-DLR and 4NSA images were compared objectively (by deriving the signal-to-noise ratios of the lateral and the medial menisci and the contrast-to-noise ratios of the lateral and the medial menisci and articular cartilages) and subjectively (in terms of the visibility of the anterior cruciate ligament, the medial collateral ligament, the medial and lateral menisci, and bone) and in terms of image noise, artifacts, and overall diagnostic acceptability. The paired t-test and Wilcoxon signed-rank test were used for statistical analyses.

**Results:**

The 1NSA-DLR images were obtained within 100 s. The signal-to-noise ratios (lateral: 3.27 ± 0.30 vs. 1.90 ± 0.13, medial: 2.71 ± 0.24 vs. 1.80 ± 0.15, both *p* < 0.001) and contrast-to-noise ratios (lateral: 2.61 ± 0.51 vs. 2.18 ± 0.58, medial 2.19 ± 0.32 vs. 1.97 ± 0.36, both *p* < 0.001) were significantly higher for 1NSA-DLR than 4NSA images. Subjectively, all anatomical structures (except bone) were significantly clearer on the 1NSA-DLR than on the 4NSA images. Also, in the former images, the noise was lower, and the overall diagnostic acceptability was higher.

**Conclusion:**

Compared with the 4NSA images, the 1NSA-DLR images exhibited less noise, higher overall image quality, and allowed more precise visualization of the menisci and ligaments.

## Introduction

Magnetic resonance imaging (MRI) plays a central role in knee evaluation and is commonly used in various clinical settings [1–3]. Acute or chronic knee pain is the principal indication for knee MRI [4, 5]. Patients in pain find it difficult to remain motionless during MRI; thus, the acceleration of the MRI scan is one of the key elements in successful knee MRI evaluation.

For the acceleration of knee MRI, various image acquisition techniques have been applied. Parallel imaging was first employed to this end [6, 7], but the disadvantages included a reduced SNR, noise enhancement, aliasing, and reconstruction artifacts [8]. Several advanced parallel imaging sequences as GeneRalized Autocalibrating Partially Parallel Acquisition (GRAPPA) and Controlled Aliasing in Parallel Imaging Results in Higher Acceleration (CAIPIRINHA) have also been applied to knee MRI overcoming some of these disadvantages [9]. Compressed sensing allows rapid MRI because it reconstructs highly undersampled k-space data and also has been successfully applied to knee MRI [10]. However, the disadvantages include image blurring and long post-processing times [11]. Finally, multislice (or multiband) imaging (the simultaneous acquisition of multiple slices) was introduced [12]. These techniques can be used in combination; recently, Del Grande et al. achieved a fourfold-accelerated 5-minute knee MRI protocol including five sequences by the combination of multislice imaging and parallel imaging [13].

Over the last decade, deep learning (DL) has found many applications (for example, in image processing, speech recognition, and natural language processing [14]). Radiologists have employed DL for image segmentation [15, 16] and lesional evaluation [17, 18]. Recently, deep learning has been applied to image reconstruction, and DL-based reconstruction (DLR) of images effectively denoised the images without compromising contrast [19–21].

We hypothesized that we could accelerate the MRI scan by using DLR without deteriorating the image quality. For routine 1.5T knee MRI, usually number of signal averages (NSA) of 2 or 3 is applied [22, 23]. Therefore, in the present study, we accelerated MRI four-fold, applied DLR, and compared objective and subjective image features with those of conventional images.

## Materials and methods

The research ethics committee of our institution (International University of Health and Welfare Chiba District Ethics Review Committee) approved the study (approval no. 20-Nr-059), and all subjects provided written informed consent. Twenty-one healthy volunteers (17 men and 4 women; mean age ± standard deviation [SD] 44.7 ± 10.9 years) were enrolled. The inclusion criteria were (1) age over 20, (2) no medical history of the knee (e.g., surgery, intra-articular injection), and (3) no apparent knee pain.

### MRI examination

All volunteers underwent 1.5-T MRI (Vantage Orian, Canon Medical Systems Corporation) using a 16-channel knee coil; proton-density-weighted images of the right knee were obtained in the coronal plane using the following parameters (repetition time 2,000 ms; echo time 33 ms; NSA 1 or 4; echo train length 8; flip angle 90°; pixel bandwidth 217 Hz; field of view 160 mm; acquisition matrix 512 × 512; slice thickness 1.5 mm; spacing between slices 2 mm; and slice number 18). Compressed sensing with parallel imaging (Compressed SPEEDER: Canon Medical Systems Corporation) was employed in this study. This technique first processes the image domain parallel imaging with random undersampling (k space undersampling rate = 64.1%) in phase encoding, followed by compressed sensing implemented with wavelet transform to remove the artifacts. The scan times were 100 s for a NSA of 1 (1NSA) and 390 s for a NSA of 4 (4NSA). The 1NSA images were subjected to DLR, yielding 1NSA-DLR images. DLR was implemented using the Advanced Intelligent Clear IQ Engine (Canon Medical Systems Corporation) [19]. Briefly, this convolutional neural network-based technique uses 7 × 7 discrete cosine transform to divide the image data into a zero-frequency component and other high-frequency components at the feature extraction layer. The former component follows a separate collateral path to maintain the image contrast, and the latter components are processed to 22 subsequent feature conversion layers for denoising. This DLR technique has also been shown to improve the image quality of cervical spine MRI [24] and diffusion-weighted whole-body imaging with background body signal suppression [25].

### Objective evaluation of image quality

**Fig. 1:**
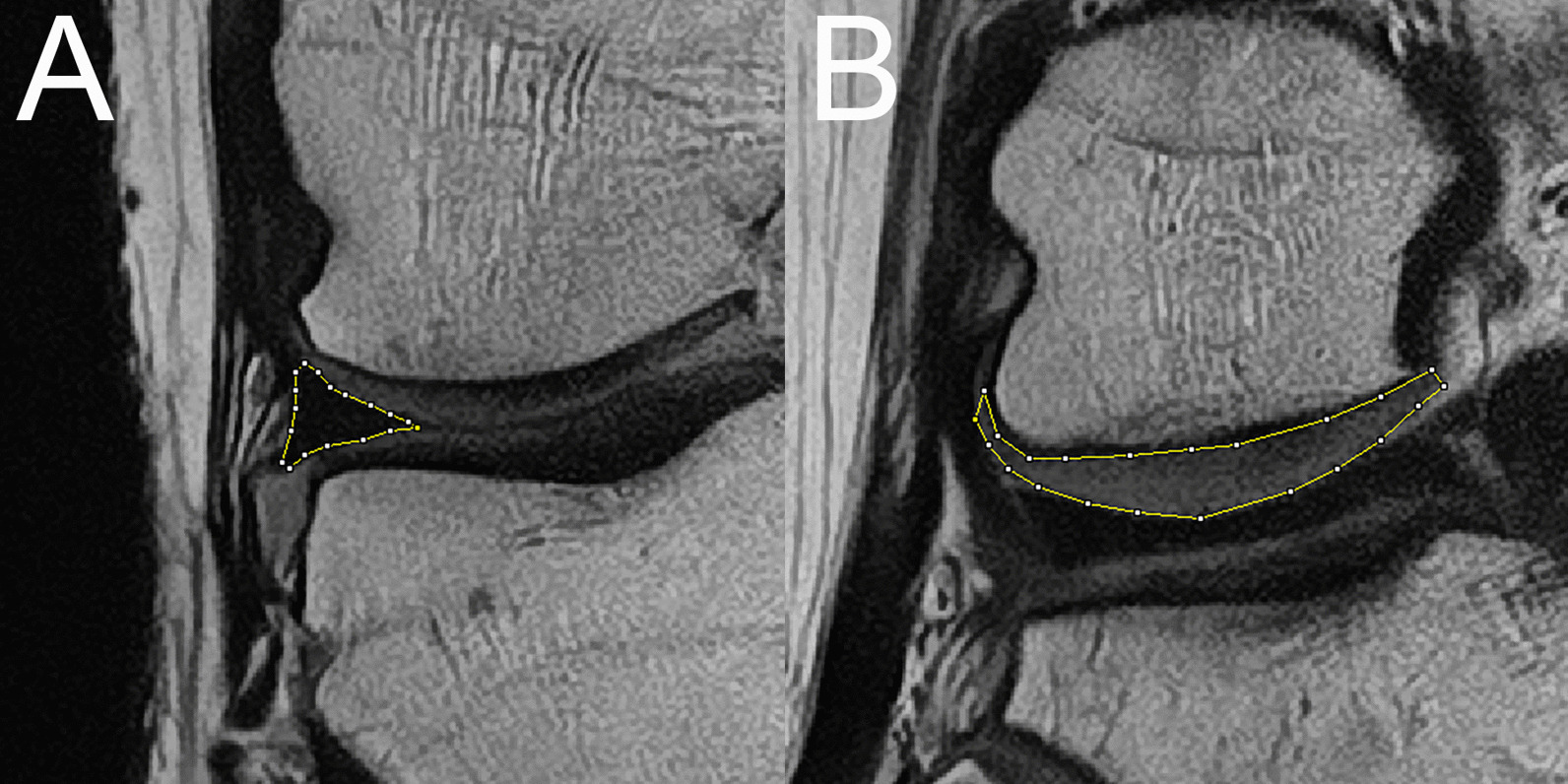
Examples of the ROIs used for subjective analysis of 1NSA-DLR image quality. **A** The middle body of the lateral meniscus. **B** The articular cartilage of the lateral femoral condyle

For the objective evaluation of image noise, SNRs of the medial meniscus (MM) and lateral meniscus (LM) were calculated. As we used a compressed sensing technique, the SNR could not be derived using the background SD. Thus, we calculated the SNR of the meniscus by employing the SD of the meniscus per se [26]. We chose meniscus as target since (1) meniscus is the one of the main structures in the knee MRI evaluation,　and (2) a structure with low signal intensity will clearly show the effect of denoising. First, we manually defined a regions of interest (ROIs) that included the entire meniscus in the slices of the middle body showing the smallest meniscus area (Fig. 1A). The SNR was calculated by dividing the mean signal intensity of the ROI by the SD of the ROI.

For objective evaluation of contrast, the contrast-to-noise ratios (CNRs) of the LM and the articular cartilage of the lateral femoral condyle and the CNRs of MM and the articular cartilage of the medial femoral condyle were calculated. We defined ROIs that covered the entire areas of cartilage in the slices with the most prominent image slice in the posterior part (Fig. 1B). This tissue-specific CNR was [26]:

$${\text{CNR}}=({\text{Mean}}_{{\rm cartilage}} - {\text{Mean}}_{{\rm meniscus}})/\sqrt({\text{SD}}_{{\rm cartilage}}^{2} + {\text{SD}}_{{\rm meniscus}}^{2}).$$

We used ImageJ software (National Institutes of Health) for quantitative image analysis. All ROIs were placed by a board-certified radiologist with 18 years of experience.

### Subjective evaluation of image quality

Three board-certified radiologists (with 17, 12, and 8 years of experience) assessed image quality. The images were presented in a random order to minimize recall bias; the observers were blinded to the acquisition method. All acquired image slices were assigned for the assessment. The visibilities of the anterior cruciate ligament (ACL), medial collateral ligament (MCL), MM, LM, and bone, as well as image noise, artifacts, and overall diagnostic acceptability, were scored using a five-point Likert scale (Table 1).

**Table 1:** The scale used for subjective image quality analysis

Grade	Visibility	Noise	Artifact	Overall
1	Not visible	Undiagnostic	Undiagnostic	Unacceptable
2	Mostly not visible or blurred	Strong noise but still diagnostic	Strong artifacts but still diagnostic	Average
3	Mostly visible but partially blurred	Acceptable noise	Acceptable artifacts	Fair
4	Subtle heterogeneity or blurring	Minimal noise	Minimal artifacts	Very good
5	Homogeneous internal intensity with sharp edges	No noise	No artifact	Excellent

### Statistical analysis

The 1NSA-DLR and 4NSA images were compared. All results are expressed as means ± SDs. The objective noises and contrasts were compared using the paired t-test because the Shapiro–Wilk test confirmed that the data were normally distributed. The subjective image qualities were compared employing the Wilcoxon signed-rank test. A *p* value < 0.05 was considered to indicate statistical significance. We evaluated interobserver agreement by calculating the Cohen weighted kappa values (quadratic weights); values of 0–0.20 indicate poor agreement, 0.21–0.40 fair, 0.41–0.60 moderate, 0.61–0.80 good, and 0.81–1.00 excellent. All analyses were performed using R software (version 4.0.5; R Foundation for Statistical Computing).

## Results

**Fig. 2:**
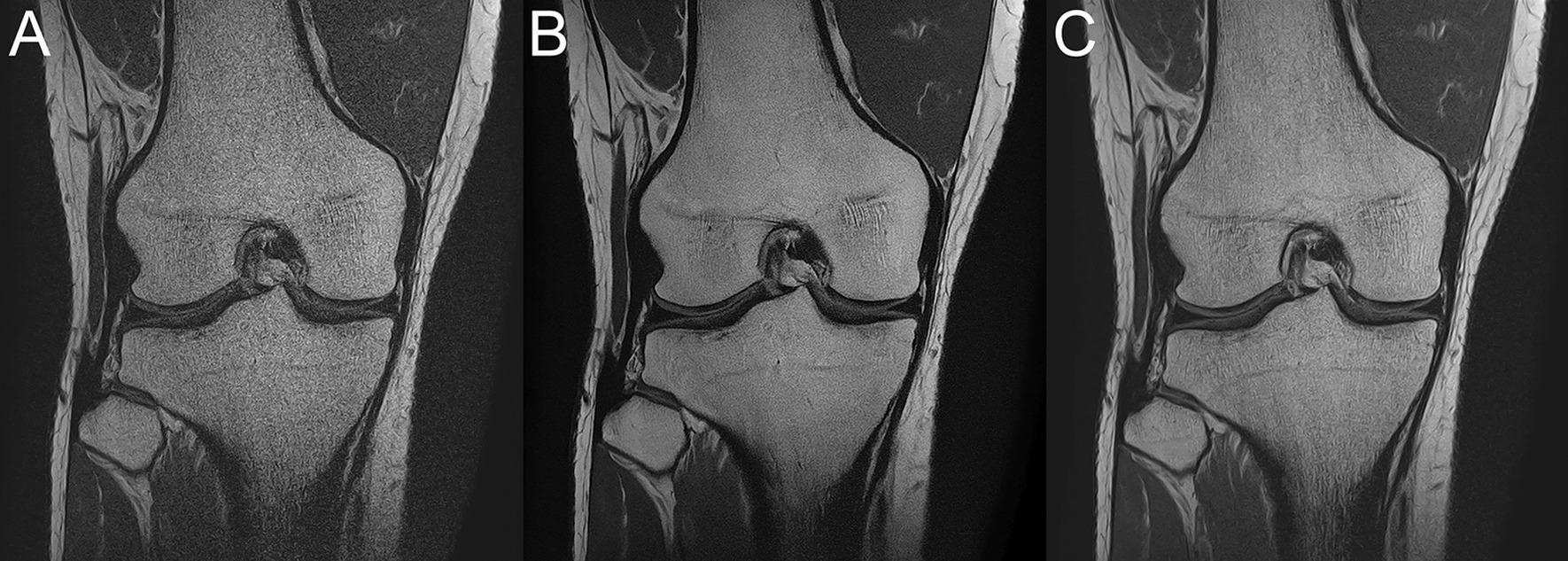
Representative MR images from a 32-year-old healthy male volunteer: **A** 1NSA image, **B** 1NSA-DLR image, **C** 4NSA image. The 1NSA-DLR image exhibits less noise compared with the 4NSA image; the overall diagnostic acceptability scores were 4 for the 1NSA-DLR image and 3 for the 4NSA image (all readers)

Representative 1NSA-DLR and 4NSA images are shown in Fig. 2.

### Objective analysis of image quality

The SNR of the LM was significantly higher in the 1NSA-DLR than in 4NSA images (3.27 ± 0.30 vs. 1.90 ± 0.13, *p* < 0.001). Similarly, the SNR of the MM was significantly higher in the 1NSA-DLR than in 4NSA images (2.71 ± 0.24 vs. 1.80 ± 0.15, *p* < 0.001). The CNR between the articular cartilage of the femoral condyle and the meniscus was significantly higher in the 1NSA-DLR than in 4NSA images in both lateral (2.61 ± 0.51 vs. 2.18 ± 0.58, *p* < 0.001) and medial sides (2.19 ± 0.32 vs. 1.97 ± 0.36, *p* < 0.001) (Table 2).

**Table 2:** The results of objective image quality analysis

	Meniscus		Cartilage		Diff(Cart – Meni)	CNR	*p* value
	Mean	SD	Mean	SD	Mean		
Lateral side							
1NSA-DLR	869.7	267.1	3029.3	797.0	2159.6	2.61 ± 0.51	**< 0.001**
4NSA	721.0	382.5	2857.7	921.7	2136.7	2.18 ± 0.58	
Medial side							
1NSA-DLR	1030.8	380.1	3440.6	1032.7	2409.7	2.19 ± 0.32	**< 0.001**
4NSA	864.6	484.7	3248.8	1124.8	2384.2	1.97 ± 0.36	

For meniscus and cartilage, mean and SD of the signal intensity of each structure is described. Diff(Cart – Meni) represents the difference in signal intensity of cartilage and meniscus. SDs are expressed as their mean and CNRs are expressed as the mean ± standard deviation. *p* values are shown for the comparison of CNR and the *p* values that indicate significant differences are shown in bold

### Subjective analysis of image quality

The results of the subjective analysis are listed in Table 3. All the observers reported that all anatomical structures except bone were better visualized on 1NSA-DLR than 4NSA images (all *p* < 0.05). Bone visualization was in fact better, and the extent of artifacts was lower in 1NSA-DLR images (all observers), but the differences were not significant (*p* > 0.05). Noise was lower (*p* ≤ 0.001) and the overall diagnostic acceptability was higher on the 1NSA-DLR than on 4NSA images (*p* < 0.01) (all observers). Cohen’s kappa analysis revealed that the extent of interobserver agreement was moderate-to-excellent in terms of structural visibilities (0.45–0.54 for ACL, 0.47–0.59 for MCL, 0.65–0.72 for MM, 0.59–0.68 for LM, and 0.66–0.85 for bone) and fair-to-excellent in terms of noise (0.32–0.61), artifacts (0.84–0.85), and overall image quality (0.60–0.77).

**Table 3:** The results of subjective image quality analysis

	ACL	MCL	MM	LM	Bone	Noise	Artifact	Overall
Reader 1								
1NSA-DLR	4.14 ± 0.35	4.90 ± 0.29	4.95 ± 0.21	5.00 ± 0.00	4.00 ± 0.00	3.71 ± 0.45	4.81 ± 0.39	3.76 ± 0.43
4NSA	3.76 ± 0.43	4.23 ± 0.68	3.95 ± 0.79	4.29 ± 0.88	3.86 ± 0.35	3.05 ± 0.21	4.42 ± 1.14	3.00 ± 0.69
*p* value	**0.03**	**0.001**	**< 0.001**	**0.005**	0.15	**< 0.001**	0.14	**0.003**
Reader 2								
1NSA-DLR	4.19 ± 0.39	4.52 ± 0.50	4.48 ± 0.50	4.48 ± 0.50	4.00 ± 0.00	3.95 ± 0.21	5.00 ± 0.00	3.95 ± 0.37
4NSA	3.71 ± 0.63	3.81 ± 0.59	3.86 ± 0.77	3.95 ± 0.72	3.81 ± 0.39	3.05 ± 0.37	4.52 ± 1.17	2.90 ± 0.61
*p* value	**0.02**	**0.001**	**0.008**	**0.02**	0.07	**< 0.001**	0.17	**< 0.001**
Reader 3								
1NSA-DLR	4.19 ± 0.39	4.62 ± 0.49	4.57 ± 0.49	4.52 ± 0.50	4.05 ± 0.21	3.95 ± 0.21	4.86 ± 0.35	3.95 ± 0.21
4NSA	3.90 ± 0.53	4.14 ± 0.77	3.86 ± 0.94	4.00 ± 0.82	3.90 ± 0.43	3.43 ± 0.58	4.43 ± 1.00	3.29 ± 0.76
*p* value	**0.04**	**0.03**	**0.01**	**0.02**	0.23	**0.001**	0.10	**< 0.001**

All variables are expressed as the mean ± standard deviation. *p* values that indicate significant differences are shown in bold

## Discussion

We found that knee MRI could be accelerated four-fold using DLR. Objectively, the 1NSA-DLR images were more uniform and showed higher CNR than the 4NSA images; subjectively, the 1NSA-DLR images revealed all studied structures more clearly than the 4NSA images, regardless of structure size. Although statistical significance was not attained, bone visibility was better and artifacts fewer on 1NSA-DLR images; noise was also significantly lower, imparting better overall image quality.

DL is relatively new, and its clinical applications are few in number. One study employed DLR to comprehensively examine the knee in 5 min without compromising image quality or diagnostic accuracy. Recht et al. performed 406 consecutive knee examinations using a 3-T MRI scanner and found that standard images and deep-learning-based accelerated images were largely equivalent [27]. This was also our experience; DLR accelerated 1.5-T knee MRI four-fold.

One of the advantages of the DLR technique is that this is a post-processing technique, so DLR can be applied to the image obtained by accelerating image acquisition techniques. In the present study, we used a compressed sensing technique in image acquisition. And by combination with DLR, clear coronal proton-density-weighted knee MR images were able to obtain within only 100s. This short acquisition time can reduce motion-related artifacts, and indeed the level of artifacts was scored better for 1NSA-DLR images in the present study. We believe that a combination of accelerating image acquisition techniques and DLR would also be beneficial for patients with knee pain.

Our work had several limitations. First, the number of volunteers was small. Second, all volunteers were recruited from one institution. Selection bias may have been in play. Third, we assessed only proton-density-weighted images because these are optimal for detecting meniscal lesions [28]. DLR is useful for processing T1- and T2-weighted images, fluid-attenuated inversion recovery images, and magnetic resonance cholangiopancreatographic images [19, 29]. Thus, DLR can effectively process other knee MRI sequences as well. Finally, we did not evaluate the disease detectability of DLR. We assume that DLR would facilitate the detection of abnormalities by radiologists, given the high image quality, but further investigations are needed.

## Conclusion

In conclusion, DLR significantly improved knee MR image quality. 1NSA-DLR images exhibited less noise, better visualization of menisci and ligaments, and higher overall image quality compared with 4NSA images.

## Data Availability

The datasets used in the present study are not publicly available due to the security of data but are available from the corresponding author on reasonable request.

## Abbreviations

ACL: Anterior cruciate ligament
CNR: Contrast-to-noise ratio
DL: Deep learning
DLR: Deep learning reconstruction
LM: Lateral meniscus
MCL: Medial collateral ligament
MM: Medial meniscus
MRI: Magnetic resonance imaging
NSA: Number of signal averages
ROI: Region of interest
SD: Standard deviation
SNR: Signal-to-noise ratio
